# Current Strategies for the Detection of Minimal Residual Disease in Childhood Acute Lymphoblastic Leukemia

**DOI:** 10.4084/MJHID.2016.024

**Published:** 2016-04-10

**Authors:** Juliana Maria Camargos Rocha, Sandra Guerra Xavier, Marcelo Eduardo de Lima Souza, Juliana Godoy Assumpção, Mitiko Murao, Benigna Maria de Oliveira

**Affiliations:** Universidade Federal de Minas Gerais – UFMG, Belo Horizonte, MG, Brazil

## Abstract

Acute lymphoblastic leukemia (ALL) is the most common cancer in children. Current treatment strategies for childhood ALL result in long-term remission for approximately 90% of patients. However, the therapeutic response is worse among those who relapse. Several risk stratification approaches based on clinical and biological aspects have been proposed to intensify treatment in patients with high risk of relapse and reduce toxicity on those with a greater probability of cure.

The detection of residual leukemic cells (minimal residual disease, MRD) is the most important prognostic factor to identify high-risk patients, allowing redefinition of chemotherapy. In the last decades, several standardized research protocols evaluated MRD using immunophenotyping by flow cytometry and/or real-time quantitative polymerase chain reaction at different time points during treatment. Both methods are highly sensitive (10^−3^ a 10^−5^), but expensive, complex, and, because of that, require qualified staff and frequently are restricted to reference centers.

The aim of this article was to review technical aspects of immunophenotyping by flow cytometry and real-time quantitative polymerase chain reaction to evaluate MRD in ALL.

## Introduction

The incidence of acute lymphoblastic leukemia (ALL) is higher in childhood and adolescence, and current treatment strategies result in long-term remission in up to 90% of children affected. However, therapeutic responses are worse in relapsed patients, indicating the value of identifying cases at high risk of relapse in order to intensify treatment and increase the survival.^1^,^2^,^3^

Diagnosis of ALL is based on the identification and quantification of lymphoblasts by the microscopic evaluation of a bone marrow (BM) sample and immunophenotypic assessment by flow cytometry, used to define the affected cell lineage and cell maturation stage and accurately classify the disease. A diagnostic approach also includes cytogenetic and molecular analyzes of a BM sample to detect chromosomal and genetic abnormalities that have prognostic and therapeutic implications.^3^,^4^–^6^

There are clinical and biological factors associated with poor prognosis in children with ALL.^3^,^7^–^10^ In the last few decades, several studies have evaluated the presence of residual leukemic cells at different times after the start of the treatment as a predictive factor for the adverse evolution of the disease. Initially, those cells were detected by morphological analysis of a BM sample, with a cutoff of 5% blast cells defining disease remission.^4^,^7^,^11^,^12^ Currently, analytical methods with better sensitivity are recommended that allow the detection of leukemic cells in lower proportions than those achieved by morphological evaluation, termed minimal residual disease (MRD).^3^,^11^,^13^,^14^ The occurrence of MRD is now considered the main prognostic indicator of ALL in children, even in patients with features that suggest a low risk of relapse, as demonstrated by many studies.^1^,^7^,^13^,^15^–^18^ MRD refines the risk stratification based on traditional features and may be used to redirect treatment.^1^,^13^,^19^ An early response to chemotherapy, with rapid reduction of neoplastic cells, especially at the end of the induction phase, is considered an important indicator of a favorable evolution and low risk of relapse.^1^,^9^,^13^,^16^,^19^–^23^

Treatment protocols for childhood ALL recommend MRD monitoring at multiple time points to evaluate the effectiveness of the various stages of chemotherapy in the elimination of leukemic cells. The evaluations carried out in the first 3 months of treatment are considered the most informative for relapse risk stratification. The absence of MRD at the end of induction therapy is considered the main favorable outcome predictor^1^,^16^,^19^,^22^ and, an even earlier MRD evaluation – within 2 to 3 weeks of the initiation of remission induction chemotherapy, has shown additional benefit in risk stratification.^15^,^24^,^25^

In a study by Conter et al. (2010), 3184 patients with B-ALL, participants of AIEOP - BFM ALL 2000 multicenter study, were stratified by MRD measured on days 33 and 78 of treatment, using RQ-PCR. Patients defined as standard risk (42%) showed a 5-year event-free survival (EFS) estimated at 92.3 %, while intermediate (52%) and high-risk patients (6%) showed a 5-year EFS of 77.6 % and 50.1 %, respectively.^1^ Basso et al. (2009) published MRD analysis results by flow cytometry on day 15 (D15) of treatment of 830 patients who underwent the same therapeutic protocol and have identified three risk groups - standard (42%), intermediate (47%) and high (11%), which showed increasing relapse incidences in five years - 7.5%, 17.5% and 47.2%. In multivariate analysis, they concluded that the assessment of MRD on D15 of treatment was the main predictor of early relapse and might complement MRD stratification in later time points.^24^ Due to the slow clearance of leukemic cells in T-ALL, Schrappe et al. (2011), when evaluating MRD by RQ-PCR in 464 children with T-lineage ALL, concluded that MRD positivity in D78 is the most important relapse risk predictor in this group of patients.^18^ Researchers of the Children’s Oncology Group (COG), analyzing data from 2143 children with B-ALL, concluded not only that MRD quantified on D29 of treatment is the most important prognostic factor for patient outcome, considering all risk groups, but also, that MRD measured in peripheral blood on D8 by flow cytometry provides additional information.^16^ Data from a study involving 99 children under 1 year indicated that the assessment of MRD (RQ-PCR for the detection of Ig/TCR genes and MLL rearrangements) allows risk stratification also in this subgroup of patients with clinical and prognostic features distinct, and can be used to redefine treatment.^23^

The accurate risk stratification using MRD evaluation requires methodologies that achieve high analytical sensitivity (10^−4^ – 10^−5^), enabling the detection of small proportions of residual leukemic cells. Less sensitive techniques (10^−2^ – 10^−3^) allow MRD detection at clinically significant levels, associated with high risk of relapse, but do not detect patients with lower levels of MRD, which also have a high risk compared to MRD-negative patients.^26^,^27^

Due to the proven association between detectable MRD and higher relapse risk, various protocols indicate the need to intensify treatment for children with detectable MRD and to reduce the intensity of chemotherapy in those who have a rapid response to treatment, with the objective of reducing toxicity.^1^,^8^,^14^,^15^,^24^,^28^,^29^

Additionally, assessing MRD offers prognostic information in patients with ALL relapse who have entered a second remission, and allows prediction of disease evolution in patients after hematopoietic stem cell transplant (HSCT).^14^,^28^,^30^ A review on this subject, published by Campana et al. (2013), after analyzing the results of several studies evaluating the MRD prognostic role in the pre- and post-HSCT periods, concluded that the risk of disease recurrence after transplantation is significantly higher among patients with detectable MRD prior to the procedure, as well as MRD detection in the post-HSCT points to an unfavorable outcome, associated with higher relapse rates.^31^ MRD detection in the pre-HSCT can also help defining strategies to improve patient outcomes, such as: establishment of the appropriate time for the procedure, choice of chemotherapy regimen, and/or use of new drug treatments that show high effectiveness in the clearance of the residual tumor cells in patients resistant to conventional chemotherapy.^31^ As an example, it should be noted the use of Blinatumomab, which represents a new class of anti-CD19 antibody-drug, that redirects T lymphocytes for selective lysis of tumor cells. A study evaluating the utilization of this drug in patients with chemotherapy-refractory ALL and an HSCT indication demonstrated its ability to eradicate resistant tumor cells in pre-transplant, and its association with higher survival rates and lower incidence of post-transplant recurrence of the disease.^32^

The prognostic value of MRD monitoring was also demonstrated in the therapeutic approach of ALL in adults, helping in the recognition of high-risk patients who have an indication for HSCT. On the other hand, those classified as true low risk, with undetectable MRD during chemotherapy induction/consolidation, may be spared from HSCT and its associated risks.^33^,^34^

## Detection Methods of MRD

The methodologies, currently available for assessing MRD, allow an average detection of one leukemic cell among 10^4^ to 10^5^ normal cells, which represents a 100-fold increase in sensitivity compared to conventional optical microscopy.^7^,^12^,^35^,^36^ Available methods include: 1) immunophenotyping of neoplastic cells by flow cytometry, which is aimed at finding cells with aberrant immunophenotypes of leukemic clones; 2) polymerase chain reaction (PCR) of the clonal rearrangement regions of T-cell receptor (TCR) and/or immunoglobulin (Ig) genes; and 3) detection of chimeric transcripts (mRNA) resulting from chromosomal translocations by reverse transcription PCR (RT-PCR).^3^,^37^–^41^

Chimeric transcripts arising from chromosomal translocations represent specific markers of leukemic clones, although only a small proportion of patients present such alterations, limiting the value of this approach.^4^,^14^,^35^,^38^ As an example, BCR-ABL fusion transcripts can be highlighted. These transcripts are present in approximately 5% of ALL in children and are considered highly relevant, due to the association of this finding with more aggressive disease, that may result in early relapse after a period of remission.^11^,^38^

In this review, the two most frequently used MRD detection methods will be covered in more detail: immunophenotyping by flow cytometry and analysis of clonal rearrangements of TCR and Ig by quantitative real-time PCR (RQ-PCR).^19^,^35^,^41^,^42^ Table 1 summarizes the main characteristics of the two methodologies, which will be addressed in the text.

The search for scientific articles was conducted in PubMed and SciELO databases, using the following keywords: minimal residual disease, acute lymphoblastic leukemia, flow cytometry, PCR, and gene rearrangements of Ig/TCR. Original and review articles published between 2005 and 2015 were initially selected and, later, relevant references cited in these items were added.

## MRD Evaluation by Flow Cytometry

The use of flow cytometry as an MRD analysis methodology emerged in the late 1980s,^43^,^44^ and its use has been increasing since then, because of further technological and methodological advances.

Immunophenotypic characterization of leukemic cells at diagnosis provides relevant information for treatment monitoring by enabling the detection of residual leukemic cells while allowing classification of the disease according to the affected cell lineage and cell maturation stage.^5^ For this purpose, it is necessary to build informative panels of monoclonal antibodies that allow an evaluation of aberrant patterns of antigen expression, including coexpression of antigens normally expressed by cells in a different maturation stage (asynchronous antigen expression); cross or aberrant expression of antigens from other cell lineages (myeloid, B-lymphoid, or T-lymphoid); and/or changes in the usual intensity of antigen expression, including overexpression, low expression, or even loss of expression.^4^,^5^,^21^,^45^,^46^ It is noteworthy that the anomalous antigen expression of blast cells reflects a genetic abnormality in the leukemic clone.^4^

MRD evaluation by flow cytometry achieves a sensitivity of 10^−3^ to 10^−4^, which is lower than the sensitivity achieved by RQ-PCR. It has the advantages of rapid turnaround time of results, which is especially important at the beginning of treatment; and broad applicability, since most ALL present identifiable leukemia-associated immunophenotypes (LAIP) at diagnosis.^4^,^14^,^16^,^20^,^33^,^42^,^47^ The main limitation of the method is associated with the phenotypic similarities between leukemic lymphoblasts and nonmalignant B-lymphocyte precursors, at the stages of bone marrow regeneration during and after chemotherapy, when false-positive results are more common. It is worth highlighting the possibility of phenotypic changes in residual leukemic cells throughout treatment, compared to the antigen expression pattern at diagnosis.^7^,^13^,^33^,^38^,^48^ The use of new cell markers and antibody panels with combinations of four or more fluorochromes is associated with improvement in sensitivity and specificity of the method.^16^,^36^,^38^,^39^,^42^ Moreover, accurate and sensitive techniques are technically and economically feasible in places with limited financial resources.^49^,^50^

MRD can be detected by flow cytometry in the early stages of remission induction chemotherapy, about two weeks after the beginning of the treatment, using a restricted panel of antibodies, since the detection of immature cells at this stage indicates the presence of residual leukemic cells.^15^,^20^,^42^,^51^

However, distinguishing between residual leukemic cells and nonmalignant B-lymphocyte precursors in samples collected in phases of chemotherapy associated with bone marrow recovery (the end of the induction phase of remission and the consolidation phase of treatment) is a challenge with this method. Prior knowledge of the standard antigen expression of lymphoid precursors in different stages of maturation and meticulous immunophenotyping of the leukemic clone at diagnosis is essential.^21^,^41^,^52^,^53^ Thus, MRD evaluation by flow cytometry at the time mentioned above points requires the use of a large antibody panel that will allow for an adequate characterization of the leukemic clone.^28^

Although there is no consensus on the panel that should be used for this purpose, different authors recommend working with strategic combinations of monoclonal antibodies conjugated with four or more distinct fluorochromes, using a relatively fixed backbone in different tubes. This strategy involves selecting three or more monoclonal antibodies that are consistently present in all of the combinations used and that define lymphoid precursor cells. One or more additional antibodies are added to each tube, in order to detect aberrant antigen expression of the leukemic clone.^4^,^13^,^21^,^24^,^25^,^29^,^36^,^47^,^48^,^50^,^54^–^56^

The core set of antibodies used to compound the backbone in the different combinations useful for MRD detection in B-lineage ALL (B-ALL) usually include CD45, CD34, CD19, and CD10 - antigen initially named CALLA (common ALL antigen), expressed with high frequency in childhood BALL.^5^,^13^,^24^,^29^,^36^,^41^,^50^,^54^,^56^ The additional antibodies should be chosen based on the immunophenotype of each case at diagnosis. Several studies have reported the applicability of different markers, among which are the following: CD123,^12^,^50^,^57^ CD58,^12^,^46^,^50^,^58^–^60^ CD38,^50^,^61^ CD66c,^46^,^62^,^63^ CD304,^36^,^64^ CD49f,^65^ CD81,^66^ and CD11b,^67^ as well as aberrant T-lymphoid or myeloid cell markers such as CD2, CD7, CD13, CD15, and CD33.^13^ Table 2 describes the antigen expression of the above-described markers that may be observed in leukemic cells and highlights the markers to be included in the backbone of the different combinations of monoclonal antibodies in an MRD detection panel in B-ALL, following the mentioned strategy.

Modulated antigen expression in the early stages of chemotherapy has been reported, including transient changes in the intensity of expression of CD10 and CD34 markers.^48^,^68^ To account for this issue, Irving et al. recommend avoiding the use of predefined gates and considering, at least, two aberrant immunophenotypes per patient.^47^

To precisely differentiate between nonmalignant B-lymphoid precursors, called hematogones, and residual leukemic precursor cells, it is important to consider the immunophenotype of normal B-cell precursors in their three different stages of maturation: early, intermediate, and late. Early B-cell precursors express the CD34 and TdT immaturity markers in combination with CD19, CD38, CD10 (bright), CD22 (weak), and CD45 (intermediate). With maturation, these cells lose their expression of CD34 and show a progressive reduction in their expression of CD10, whereas they gain CD20, CD22, and CD45 expression. Surface immunoglobulin acquisition coincides with the gain of CD20 expression. Mature B-lymphocytes show bright expression of CD22 and CD45, reduced or no expression of CD38, and no expression of CD10. On the other hand, leukemic lymphoblasts are characterized by the overexpression of CD10 and reduced or no expression of CD45, in addition to the asynchronous expression of early and late antigens, and aberrant expression of other markers.^6^,^12^,^52^,^53^,^69^

The immunophenotype of malignant T-lymphoblasts for the most differs significantly from that of normal marrow and blood T-cells, allowing easier detection of MRD. The identification of immature T-cells in the peripheral blood (PB) or BM of T-cell ALL (T-ALL) patients indicates MRD, as only cells confined to the thymus (thymocytes) should show these characteristics.^34^,^65^ Table 3 presents commonly used markers in the MRD analysis of T-ALL samples, including CD3cy (cytoplasm), CD3s (surface), CD7, CD34, TdT, and CD99, with a description of the antigen expression changes that are observed.^5^,^7^,^12^,^24^,^28^,^46^,^55^,^70^ The panel could also include other markers of T-lineage cells, such as CD1a, CD2, CD4, CD5, and CD8, and/or aberrant B-lymphoid and myeloid markers, such as CD19, CD13, and CD33, depending on the phenotype determined at diagnosis.^28^,^49^,^70^

In order to allow appropriate determination of residual leukemic clones, the characterization of a cluster of at least 10 events within a given sample is recommended. Thus, to achieve a sensitivity of 1 × 10^−4^, corresponding to the detection limit of 0.01%, analysis of a minimum of 10^5^ leukocytes is necessary.^4^,^7^,^21^,^25^,^29^,^51^,^56^ The proportion of blast cells should be determined among the total viable nucleated cells, marked with the nuclear dye SYTO, thus eliminating the inclusion of anucleated events like erythrocytes, platelets, and debris.^25^,^48^,^51^,^56^,^68^

The evaluation of MRD should preferably be performed with BM samples. MRD levels show a good correlation in PB and BM in T-ALL; however, the correlation is weak in B-ALL, with lower levels of MRD in PB.^49^,^71^

The preparation of PB or BM samples for analysis might include mononuclear cells isolation by centrifugation gradient using Ficoll-Hypaque solution.^15^,^36^,^45^,^51^,^62^ However, the processing of whole blood samples or whole BM has been the procedure of choice in several studies as it presents the following advantages: it prevents the selection or arbitrary loss of specific cell populations; it enables the reliable enumeration of cell populations present in the sample; it minimizes the chances of modification of antigen expression; and it reduces the processing time of the samples.^4^,^20^,^37^ A study by Luria et al.,^51^ comparing these two ways of sample processing, revealed high correlation coefficients in samples collected on days 15 (0.875) and 33 (0.82) of treatment. Gaipa et al.^41^ obtained a similar result in an analysis of 266 samples collected on days 15, 33, and 78 of treatment, with 91% concordant results between the mononuclear cell and total nucleated cell preparations, using a cutoff value of 0.01%. Higher sensitivity was observed in the analysis of mononuclear cells, which was attributed to the greater number of cells acquired.^29^ Irving et al. found a concordance rate of 86% between molecular methods and flow cytometry in MRD detection in BM samples processed in different ways, including mononuclear cell preparations for molecular methods and red blood cells lysis in whole BM samples for immunophenotyping by flow cytometry.^47^

A study conducted by Dworzak et al., which was aimed for interlaboratory standardization of flow cytometry assays for MRD detection at multiple time points in treatment, showed a high concordance of results obtained in an analysis of 202 samples from four participating centers, with concordant results in 76% (four centers) and 96% (three centers) of the samples.^56^ In order to understand the discordant results, technical difficulties inherent to the analysis of samples with normal lymphoid regeneration, low MRD levels (levels close to the detection limit) and technical flaws (contamination of tubes and compensation failures, for example) were considered. Additionally, Luria et al., in a comparative analysis of the results of two different laboratories, highlighted the importance of the standardization of data analysis procedures, revealing that half of the discrepancies between results could be explained by variations in data interpretation.^51^

## Analysis of Clonal Rearrangements of the Ig and TCR Genes by RQ-PCR

Antigen receptor genes (Ig and TCR) include various discontinuous segments (regions V, variable; D, diversity; and J, junction), which undergo variable rearrangements during early stages of B and T-lymphoid cell differentiation. Ig and TCR diversity is generated by a random joining of a V(D)J exon. During this process, the deletion and/or random insertion of nucleotides at segment’s junctions can also occur by forming the so-called N regions. Thus, the regions resulting from Ig and TCR gene rearrangements represent sequences that are unique to each lymphocyte.^11^,^21^,^35^,^72^–^74^ In leukemia, Ig and TCR rearrangements can occur in either B or T-cells. Therefore, rearrangements of the immunoglobulin heavy chain gene (IgH), light chain kappa (IgK), TCR delta (TCRD), TCR gamma (TCRG), TCR beta (TCRB) and light chain lambda (IgL) may be detected at different frequencies in ALL of B and T-cell lineages.^11^,^17^,^19^,^35^,^37^,^41^,^62^,^68^,^72^–^79^

An analysis of clonal rearrangements of Ig and TCR genes by PCR, at diagnosis, aims at finding specific sequences of leukemic clones, usually present in ALL of T and precursor B-cells, which can be used as a target in MRD evaluation.^11^,^72^ The sensitivity of PCR assays can vary, depending on the identified gene rearrangement regions, on the use of specific primers for individual V, D, and J regions or consensus primers for conserved regions, on the total amount of DNA analyzed, on the background identified in normal lymphoid cells, and on the methodological approach used.^11^,^72^,^80^

Conventional PCR methods, developed in the 1990s, require post-PCR detection techniques (electrophoresis or dot blotting and hybridization) to identify the final products of the amplification reaction.^38^,^72^ As an example, the amplified clonal rearrangements using consensus primers can be identified based on the size and signal intensity after electrophoretic separation and subsequent heteroduplex analysis, to distinguish PCR products derived from monoclonal and polyclonal lymphoid cells. In follow-up samples, the electrophoretic profiles obtained are compared with those found at diagnosis. This conventional technique shows a maximum sensitivity of 0.1%. However, from a methodological point of view, it is considered relatively simple, fast and low-cost. Although it does not identify residual leukemic cells in proportions lower than 10^−3^, it allows the identification of patients with greater residual tumor burdens and those at high risk of relapse, and can be considered a cost-effective methodology for MRD monitoring in countries with limited financial resources.^27^,^79^ A qualitative MRD result (presence or absence) provides limited information and does not allow for an evaluation of tumor kinetics, making it impossible to correlate the final amount of PCR product and the initial amount of target molecules.^11^

The RQ-PCR technique represents a significant advance, as it allows the accurate quantification of a PCR product during the early exponential phase of the amplification reaction, eliminating the variability of the late exponential phase and the need for post-PCR manipulation.^35^,^38^,^72^ RQ-PCR methods require the design of primers specific for each patient and, therefore, the additional step of sequencing the clonal rearrangement identified at diagnosis, and detection of the signal in follow-up samples is considered specific for the malignant clone.^11^ The detection of Ig and TCR gene rearrangements by RQ-PCR is currently considered the gold standard for MRD assessment in ALL.^46^ It has the advantages of high analytical sensitivity (10^−4^ to 10^−5^), use of standardized methods, and applicability to most patients with the disease.^17^,^19^,^35^,^37^,^38^,^39^,^42^,^76^,^80^ Disadvantages include the high cost; the difficulty in providing fast results, due to the time required to design clone-specific primers; and the possibility of false-negative results due to oligoclonality or new gene rearrangements during disease.^14^,^17^,^35^,^38^ It is noteworthy that the use of this technique may be restricted to specialized laboratories, due the complexity of the analyses.^33^,^35^,^76^

In the context of the analysis of Ig and TCR gene rearrangements by PCR methods, it is important to highlight the relevance of the BIOMED-2 Concerted Action – a European collaborative study, conducted by van Dongen et al., that has developed and standardized PCR primer sets for the detection of the gene rearrangements. Since the completion of the study, all primers and multiplex tubes are available on a commercial basis.^73^

In addition to Ig and TCR gene rearrangements, genomic breakpoints that are secondary to specific translocations, such as rearrangements of the MLL gene or SIL-TAL fusion genes, represent alternative DNA targets, although they are less frequently seen.^17^,^23^,^38^,^75^

## Summary of the Technical Recommendations for RQ-PCR

Although BM samples are recommended for the analysis of Ig and TCR clonal rearrangements by RQ-PCR, PB yields comparable results in T-ALL.^38^,^81^ The tests should be performed on mononuclear cells separated by centrifugation gradient, using Ficoll-Hypaque solution, which increases the sensitivity and reproducibility of the method.^38^,^75^

For RQ-PCR analyses, standard procedures are described in the literature,^17^,^33^,^35^,^37^,^38^,^68^,^72^,^73^,^75^,^76^,^80^ and recommendations for each step are as follows: 1) DNA amplification by conventional PCR using consensus primers for the search of Ig and/or TCR gene rearrangements; 2) Detection of the PCR product by polyacrylamide gel electrophoresis; 3) Heteroduplex analysis (or gene scanning) for the differentiation of PCR products derived from monoclonal and polyclonal lymphoid cells,^82^ followed by excision and elution of the band from the polyacrylamide gel if a homoduplex within the expected size range is confirmed; 4) Sequencing of the junction regions of the rearrangements; 5) Comparison to known sequences obtained from available electronic databases for the identification of the V, D, J segments involved, and the identification of the N region; 6) Design of clone-specific primers for the junctional regions; 7) Execution of RQ-PCR using specific primers for each patient and standard curves generated from serial dilutions (10^−1^ to 10^−5^) of the sample collected at the initial diagnosis in a DNA pool of mononuclear cells obtained from 5 to 10 healthy donors, tested in replicates; 8) Detection of the reaction products by nonspecific systems (dyes, e.g., SYBR Green I) or specific systems (hydrolysis probes or hybridization probes conjugated to fluorochromes); 9) Analysis of the fluorescent signal obtained, based on the fluorescence intensity of the background, often determined during the first three to 15 PCR cycles – parameter used to calculate the cycle threshold (C_T_) of each sample (the PCR cycle at which the fluorescence exceeds the cutoff for the first time); and 10) Correction for the amount and quality of DNA by the amplification of control genes in parallel with the test sample.

Highly sensitive RQ-PCR assays require accurate identification of the sequences of the junctional regions of Ig and TCR clonal rearrangements in each case, which allows the design of specific oligonucleotides.^38^ The specificity of the reaction is assessed by parallel amplification of a DNA pool control sample obtained from healthy donors. The sensitivity is defined based on dilution assays that can be performed with the diagnostic sample or reference materials. The limit of detection is determined by the last dilution able to generate a positive signal in the absence of a signal of the polyclonal DNA control sample, and that can detect each junctional region identified as a target within the reproducibility range or quantitative range of the test.^38^,^72^,^75^,^80^ In an evaluation of the reproducibility, the variation in the C_T_ values of the replicates should be less than 1.5 if the average C_T_ value of the replicates is less than 36. It may be higher if the average C_T_ value of the replicates is greater. In the case of nonspecific amplification, the difference in C_T_ values between specific and nonspecific amplifications must be at least one cycle, although preferably greater than or equal to three, to minimize false positive results.^72^,^75^,^80^ The standard curve, obtained from at least three dilutions, must present an acceptable slope (between 3.1 and 3.9) and correlation coefficient (>0.98), according to van der Velden et al.^80^ The quantitative range and the analytical sensitivity of the test must be determined for the RQ-PCR reaction of the diagnostic sample to establish the parameters for follow-up samples from the same patient. If follow-up samples present MRD results out of the quantitative range of the test, the data should be considered non-reproducible and, therefore, unable to generate accurate quantitative results.^80^ To interpret the results adequately, the laboratory report should specify the quantitative range and the analytical sensitivity of the test.^80^

False positive results from PCR reactions may be due to the presence of contaminating DNA or non-specific hybridization to amplified DNA from normal lymphocytes. False negative results may depend on oligoclonality or clonal evolution during the disease, leading to the loss of targets identified at diagnosis.^11^,^72^ Therefore, the use of at least two Ig/TCR targets per patient is recommended for greater accuracy of MRD tests.^17^,^35^,^37^,^38^,^72^,^80^

Due to the technical complexity, RQ-PCR assays for MRD detection in ALL should be performed by reference molecular hematology laboratories that regularly participate in external quality control programs and preferably carry out the analysis for a significant number of new cases per year (minimum of 50 cases).^38^,^80^

## Comparative Studies Between Molecular Techniques and Flow Cytometry

MRD detection by flow cytometry and/or PCR techniques has been widely used in studies of childhood ALL. Publications report MRD evaluations using techniques with different analytical sensitivities, follow-up samples collected at various times, and groups of patients submitted to different treatment protocols.^1^,^13^,^15^,^16^,^19^,^20^,^22^–^24^,^35^,^42^,^54^,^83^ On the other hand, all of these studies confirm the value of detecting small numbers of residual leukemic cells to assess the risk of disease relapse and determine the chemotherapy regimen.

Comparative studies have shown that MRD analyses by flow cytometry and RQ-PCR methodologies estimate similar levels of residual leukemic cells in most samples obtained from children with ALL, when present in amounts greater than 0.01%.^14^,^19^,^29^,^37^,^47^,^62^,^84^ With the current techniques, samples with residual leukemic cells detected by RQ-PCR at levels below 0.01% are often negative by flow cytometry.^28^,^29^ Thus, concordance between the results obtained by the two methods may depend on the cutoff used and on the evaluation time.^29^,^37^,^84^ Gaipa et al., in a simultaneous analysis of 3,565 BM samples by both methods, at day 15, day 33, and day 78, found a general concordance rate of 80% using a cutoff value of 0.01%.^29^ However, in an evaluation of the times of sample collection, concordance between the results obtained at day 33 was lower (70%) than at days 15 and 78 (86% and 87%, respectively). The discordant results were most often negative by flow cytometry and positive by RQ-PCR, in samples with low MRD levels (< 0.1%). Using RQ-PCR as a reference, the sensitivity of flow cytometry was 87% at day 15, decreasing to 47% at day 33 and to 27% at day 78, due to the progressive reduction in MRD levels during follow-up of patients. The specificity of flow cytometry was high at all three-time points: 74% at day 15, 88% at day 33, and 99% at day 78. Similarly, Mejstríková et al., using a strategy of “predefined gates” observed a higher concordance between the two methodologies at day 15, when compared to day 33 and to week 12 of treatment.^46^ Malec et al. described similar results for an analysis of 71 follow-up samples, with 89% concordance between the two methods, using a cutoff value of 0.01%.^37^ Kerst et al. observed an even higher concordance (97.1%) in a comparative analysis of 105 follow-up samples, with no indication of the collection times.^62^ Using the same cutoff value, Ryan et al. observed a qualitative concordance between the methods in 93.8% of 151 samples analyzed at multiple collection time points over a period of three years. However, the concordance of the results at day 28 (25 samples) and in the consolidation phase (weeks 7 to 12, 17 samples) was lower (84% and 88%, respectively).^84^ In order to explain the false RQ-PCR results, the authors considered the inability to distinguish between viable and apoptotic cells and the possibility of subclone emergence. In a view to explain the false results by flow cytometry, they examined the difficulty of a phenotypic distinction between leukemic cells and normal B-precursors of the regenerating BM, in addition to the possible modulation of antigen expression during treatment.

## Novel Technologies

A very sensitive sequencing assay, recently developed and described by Faham et al., allows the detection and monitoring of all leukemic rearrangements in a given sample, enabling the detection of clonal evolution in follow-up samples and reducing false negative results.^85^ This methodology is based on next-generation sequencing (NGS) and uses consensus primers to amplify all Ig and TCR rearrangements present in the leukemic clone at diagnosis, allowing their monitoring during treatment. NGS shows an analytical sensitivity of 10^−6^, higher than that achieved by RQ-PCR, and do not demand specific primer design for each patient, requiring less time to execute. On the other hand, it has disadvantages such as high complexity and cost.

## Conclusions

Despite the extensive literature already available, it is necessary to evaluate data on MRD as a prognostic factor in ALL for each therapeutic regimen, considering the differences in the intensity of treatment protocols, favorable times for evaluation and methodological differences among the assays.^20^,^33^,^35^,^83^,^86^

It is recommended that MRD cutoffs for therapeutic decision are defined within each treatment protocol for ALL, since they depend on the detection method, the treatment administered before MRD follow-up, the prognostic stratification of patients and the protocol treatment goals.^33^,^38^ The cutoff value used by most studies to define MRD positivity is 0.01%, which is the detection limit of routine tests.^14^,^19^,^29^ The recent introduction of high-sensitivity techniques might change the cutoff point for risk stratification in the near future if very low levels of MRD are proven to be of clinical value.

If properly standardized, immunological and molecular methods are equally effective in the detection of clinically significant levels of MRD.^7^,^14^,^19^,^29^,^37^,^47^,^62^,^83^,^84^ In general, PCR-based methods are considered more laborious than immunological methods, and they may have the additional difficulty to rapidly design clone-specific primers for early MRD analyses.^42^,^50^

The detection of residual leukemic cells by flow cytometry in stages of treatment associated with bone marrow regeneration requires more complex technical validation to achieve results comparable to those obtained with the RQ-PCR. Thus, it is possible to use flow cytometry as an alternative or a complement to the molecular method in monitoring patients undergoing treatment for ALL.

As an example, it is worth mentioning the strategy defined by Coustan-Smith et al., who chose to use flow cytometry to monitor MRD during remission induction therapy, reserving Ig and TCR rearrangements amplification assays for inconclusive cases.^28^ Other authors also suggest the complementary use of the two methodologies, whenever possible and economically feasible, to accurately stratify patients by MRD and prevent false negative results due to clonal evolution or phenotypic changes.^1^,^7^,^13^,^14^,^29^,^62^,^84^,^86^

MRD monitoring during chemotherapy treatment of childhood ALL is recommended by guidelines adopted by the main reference institutions of onco-hematology, even in areas with limited technical and financial resources. MRD detection by flow cytometry is a viable alternative for services located in such regions. In contrast, analysis of Ig and TCR gene rearrangements by RQ-PCR is considered an expensive method, which can limit its use. Alternatively, some authors have proposed the detection of Ig/TCR rearrangements by conventional PCR using consensus primers and homo/heteroduplex analysis, despite its lower analytical sensitivity, considering that this approach allows identification of patients with greater residual tumor burden, and then at high risk of relapse.^22^,^27^,^49^,^50^,^78^,^79^,^87^,^88^

On the other hand, technological development incorporated by laboratories in regions with greater resources can facilitate and enhance the assessment of MRD. Thus, methodologies such as next-generation sequencing and multiparameter flow cytometry (≥ 8 colors) with automation of data analysis tend to replace progressively currently available methods.^26^,^86^

## Figures and Tables

**Table 1:** Characteristics of the two most frequently used MRD detection methods*

	Detection of aberrant immunophenotypes by Flow Cytometry	Analysis of clonal rearrangements of *Ig* and *TCR* genes by RQ-PCR
Analytical sensitivity	10^−3^ – 10^−4^	10^−4^ – 10^−5^
Applicability	> 90% of patients	> 90% of patients
Advantages	- Rapid turnaround time of results - Allows early MRD analyses - Ability to distinguish between viable and apoptotic cells - Relatively less expensive	- Standardized methods - High sensitivity
Disadvantages	- False positive results due to phenotypic similarities between leukemic lymphoblasts and regenerating B-lymphocyte precursors - False negative results due to phenotypic changes in residual leukemic cells throughout treatment - Limited standardization	- High cost - Technical complexity - Difficulty in providing fast results - Difficulty to rapidly design clone-specific primers for early MRD analyses - False negative results due to oligoclonality or clonal evolution - Inability to distinguish between viable and apoptotic cells

Ig, immunoglobulin; TCR, T-cell receptor; RQ-PCR, real time quantitative polymerase chain reaction; MRD, minimal residual disease.

* Based on van Dongen et al.,^26^ Scrideli et al.,^27^ Campana et al.,^31^ Schrappe.^40^

**Table 2:** Examples of markers used in MRD detection by flow cytometry in B-ALL, with a description of the antigen expression expected pattern and/or possible anomalous antigen expression of blast cells, in relation to the usual antigen expression of normal B-cell precursors.

Marker	Antigen expression noted in blast cells
CD45*	Reduced expression or eventually absent
CD34*	Frequently present (immaturity cell marker)
CD10*	Frequently present and overexpressed in childhood B-ALL
CD19*	Maintained expression (cell lineage marker)
CD11b	Aberrant expression
CD38	Reduced expression
CD49f	Increased expression (overexpression)
CD58	Increased expression (overexpression)
CD66c**	Aberrant expression
CD81	Reduced expression
CD123	Increased expression (overexpression)
CD304	Aberrant expression

MRD, Minimal Residual Disease; B-ALL, B-acute lymphoblastic leukemia.

* Backbone markers to be included in different combinations of monoclonal antibodies in an MRD detection panel in B-ALL.

** The most frequently aberrant myeloid antigen observed.

**Table 3:** Commonly used markers in the MRD analysis by flow cytometry in T-ALL, with a description of the aberrant antigen expression possibly noted.

Marker	Antigen expression noted in blast cells
CD3 cytoplasm	Maintained expression (cell lineage marker)
CD3 surface	Reduced expression or absence
CD7	Maintained expression (cell lineage marker)
CD34	Frequently present (immaturity cell marker)
TdT	Nuclear expression frequently present
CD99	Overexpression in thymic immature T-cells and T-lymphoblasts; weak expression or absence in circulating T-lymphocyte

TdT, terminal deoxyribonucleotidyl transferase
